# LRRK2-Related Parkinson’s Disease Due to Altered Endolysosomal Biology With Variable Lewy Body Pathology: A Hypothesis

**DOI:** 10.3389/fnins.2020.00556

**Published:** 2020-06-04

**Authors:** Pilar Rivero-Ríos, María Romo-Lozano, Rachel Fasiczka, Yahaira Naaldijk, Sabine Hilfiker

**Affiliations:** ^1^Institute of Parasitology and Biomedicine “López-Neyra”, Consejo Superior de Investigaciones Científicas (CSIC), Granada, Spain; ^2^Life Sciences Institute, University of Michigan, Ann Arbor, MI, United States; ^3^Department of Anesthesiology, New Jersey Medical School, Rutgers, The State University of New Jersey, Newark, NJ, United States

**Keywords:** LRRK2, Parkinson’s disease, Rab protein, lysosome, phosphorylation, α-synuclein, glucocerebrosidase, lysosomal storage disorder

## Abstract

Mutations in the gene encoding for leucine-rich repeat kinase 2 (LRRK2) are associated with both familial and sporadic Parkinson’s disease (PD). LRRK2 encodes a large protein comprised of a GTPase and a kinase domain. All pathogenic variants converge on enhancing LRRK2 kinase substrate phosphorylation, and distinct LRRK2 kinase inhibitors are currently in various stages of clinical trials. Although the precise pathophysiological functions of LRRK2 remain largely unknown, PD-associated mutants have been shown to alter various intracellular vesicular trafficking pathways, especially those related to endolysosomal protein degradation events. In addition, biochemical studies have identified a subset of Rab proteins, small GTPases required for all vesicular trafficking steps, as substrate proteins for the LRRK2 kinase activity *in vitro* and *in vivo*. Therefore, it is crucial to evaluate the impact of such phosphorylation on neurodegenerative mechanisms underlying LRRK2-related PD, especially with respect to deregulated Rab-mediated endolysosomal membrane trafficking and protein degradation events. Surprisingly, a significant proportion of PD patients due to LRRK2 mutations display neuronal cell loss in the substantia nigra pars compacta in the absence of any apparent α-synuclein-containing Lewy body neuropathology. These findings suggest that endolysosomal alterations mediated by pathogenic LRRK2 *per se* are not sufficient to cause α-synuclein aggregation. Here, we will review current knowledge about the link between pathogenic LRRK2, Rab protein phosphorylation and endolysosomal trafficking alterations, and we will propose a testable working model whereby LRRK2-related PD may present with variable LB pathology.

## Introduction

Parkinson’s disease (PD) is a progressive neurodegenerative disorder affecting 1% of the population above the age of 60 years, and 5% of individuals over the age of 85 (Reeve et al., 2014). It is generally characterized by two major neuropathological hallmarks including neuronal loss, which mainly affects dopaminergic (DA) neurons in the *substantia nigra pars compacta* (SNpc), and the presence of proteinaceous inclusions known as Lewy bodies (LBs) and Lewy neurites rich in α-synuclein in many of the surviving neurons. The loss of these DA neurons results in the classical motor symptoms of PD, including shaking, rigidity, and slowness of movement (Kalia and Lang, 2015).

The molecular events leading to the loss of DA neurons are not well understood, and their identification is complicated by the fact that around 90% of PD cases are sporadic, meaning that there is no apparent underlying cause. However, the identification of monogenic forms of PD, in which autosomal-dominant, or autosomal-recessive mutations in certain genes cause the disease with variable penetrance is of great importance to PD research, as it allows for the generation of cellular and animal models carrying the mutations to study the mechanisms implicated in the disease (Reed et al., 2019).

Point mutations in the leucine-rich repeat kinase 2 (LRRK2) gene are the most frequent cause of familial, autosomal-dominant Parkinson’s disease (PD; Brice, 2005; Di Fonzo et al., 2006; Lesage et al., 2006; Ozelius et al., 2006), and sequence variations in LRRK2 are known to modify PD risk, indicating that it also plays a role in the most common sporadic form of the disease (Gilks et al., 2005; Nalls et al., 2014). In addition, patients with LRRK2 variations present with late-onset disease and core clinical features indistinguishable from sporadic PD (Ren et al., 2019). As a result, LRRK2 has become the subject of intense studies to understand some of the cellular processes that contribute to disease pathogenesis.

**FIGURE 1:**
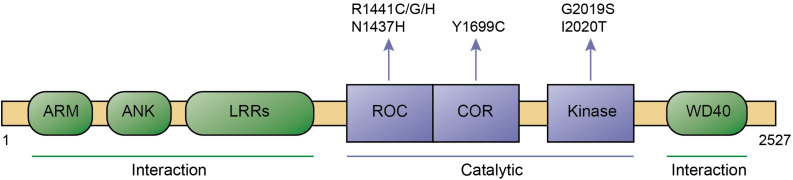
Domain structure and pathogenic mutants of LRRK2. Domains, pathogenic mutations, interaction regions, and catalytic regions are as indicated. ARM, Armadillo; ANK, Ankyrin; LRRs, Leucine-rich repeats; ROC, Ras of complex; COR, C-terminal of ROC; and WD40, WD40 repeat domain.

Leucine-rich repeat kinase 2 is a large protein which belongs to the ROCO protein family, characterized by the presense of a ROC (Ras-of-complex) GTPase, a COR (C-terminal of ROC), and a kinase domain. Apart from such catalytic core, it contains a series of protein-protein interaction domains including N-terminal armadillo, ankyrin and leucine-rich repeats, as well as C-terminal WD40 repeats (Figure 1). Over 100 LRRK2 variants have been described, and a small set of those have been shown to be pathogenic, including R1441C/G/H and N1437H in the ROC domain, Y1699C in the COR domain, and G2019S and I2020T in the kinase domain, respectively (Islam and Moore, 2017; Figure 1). The G2019S mutation is the most common, and has been found in both familial and sporadic PD cases. In contrast to all other pathogenic LRRK2 mutations which are highly penetrant, the G2019S variant displays significantly reduced penetrance which increases with age (Goldwurm et al., 2007; Gasser, 2015; Christensen et al., 2018). This is consistent with the idea that PD can be attributed to a combination of genetic, environmental and age-related factors, and indicates that the G2019S LRRK2 variant serves as an ideal model system to investigate mechanisms underlying sporadic PD pathogenesis (Ren et al., 2019).

Whilst only the G2019S LRRK2 mutation seems to increase LRRK2 kinase activity when assayed *in vitro* (West et al., 2005, 2007; Gloeckner et al., 2006; Greggio et al., 2006, 2007; Smith et al., 2006; Guo et al., 2007; Iaccarino et al., 2007; Jaleel et al., 2007; Lewis et al., 2007; Luzón-Toro et al., 2007; Imai et al., 2008; Anand et al., 2009; Covy and Giasson, 2009; Greggio and Cookson, 2009), all pathogenic LRRK2 mutants converge on enhancing LRRK2 kinase substrate phosphorylation when assayed *in vivo* (Steger et al., 2016). Highly potent, selective and brain-permeable kinase inhibitors have been developed and are in various stages of clinical development (West, 2017). At the same time, extensive research efforts are under way to gain detailed knowledge of the cellular deficits mediated by pathogenic LRRK2, as this may point toward additional targets for disease-modifying therapeutics.

A multitude of studies indicate that pathogenic LRRK2 alters vesicular trafficking events which all ultimately impact upon proper endolysosomal functioning (MacLeod et al., 2006, 2013; Plowey et al., 2008; Alegre-Abarrategui et al., 2009; Gómez-Suaga et al., 2012, 2014; Beilina et al., 2014; Yakhine-Diop et al., 2014; Henry et al., 2015; Hockey et al., 2015; Saha et al., 2015; Eguchi et al., 2018; Schapansky et al., 2018; Korecka et al., 2019; Rivero-Ríos et al., 2019; Wallings et al., 2019). Phosphoproteomic studies have identifed a subset of Rab proteins including Rab1, Rab3, Rab5, Rab8, Rab10, Rab12, Rab29, Rab35, and Rab43 as physiological substrates for LRRK2 (Steger et al., 2016; Thirstrup et al., 2017; Jeong et al., 2018; Taylor and Alessi, 2020). Since Rab GTPases are key players of all intracellular vesicular trafficking steps (Wandinger-Ness and Zerial, 2014), this raises the possibility that pathogenic LRRK2 may cause such endolysosomal deficits by impairing the functioning of one or several of these Rab substrates. The lysosome is key for the degradation of a large variety of proteins, protein aggregates, and defunct organelles. Therefore, aberrant lysosomal functioning mediated by pathogenic LRRK2 may be expected to cause the buildup of protein aggregates including α-synuclein, yet a significant portion of LRRK2-related PD cases do not display any apparent LB pathology. Here, we will summarize current knowledge about the link between pathogenic LRRK2 activity, Rab phosphorylation and concomitant lysosomal deficits, which may contribute to cell death associated with LRRK2-related PD in either the absence or presence of α-synuclein pathology.

## Protein Aggregation in Sporadic and Familial PD

The presence of proteinaceous inclusions caused by the aggregation of misfolded proteins in distinct regions of the brain is a common neuropathological feature of many neurodegenerative disorders. Tau is a microtubule-binding protein which accumulates as neurofibrillary tangles in the brains of patients with Alzheimer’s disease (AD) as well as other neurodegenerative diseases including sporadic PD (Zhang et al., 2018). Intracellular aggregates of α-synuclein are common to all α-synucleinopathies and are called LBs in PD and in Dementia with Lewy Bodies (DLB), glial cytoplasmic inclusions in multiple system atrophy (MSA), and axonal spheroids in several less well-characterized neuroaxonal dystrophies (Kim et al., 2014). Alpha-synuclein pathology is observed in over 50% of autopsy-confirmed AD brains (Twohig and Nielsen, 2019), and both α-synuclein and AD-related pathology can be detected in up to 25% of cognitively healthy elderly subjects (Markesbery et al., 2009). The observed pathology in healthy individuals may reflect a presymptomatic disease stage, or the existence of diverse α-synuclein strains not equally toxic to cells (Peng et al., 2018). In either case, these findings indicate that neither LB nor tau pathology are specific to a given disease entity.

**FIGURE 2:**
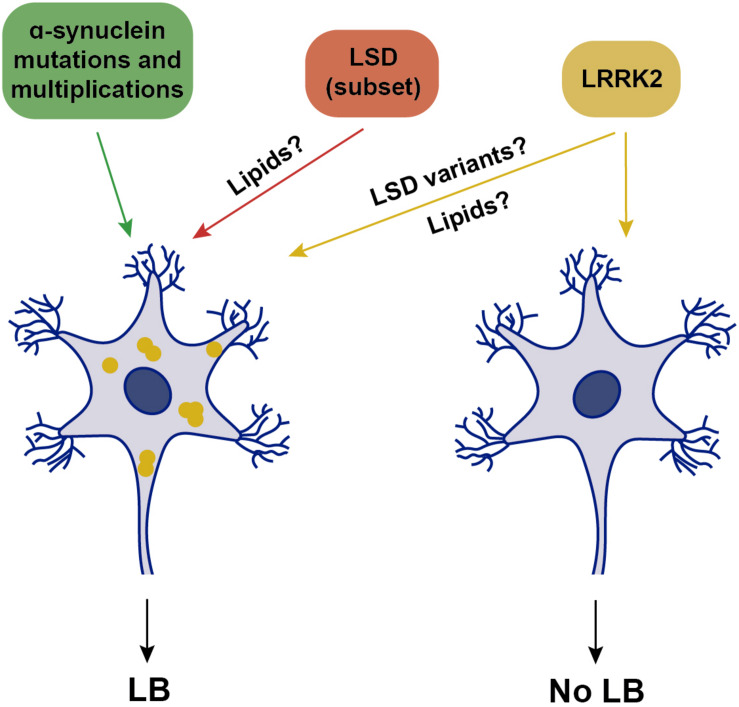
Schematics of genetic alterations and their association with LB pathology. Mutations or multiplications in α-synuclein are associated with LB pathology, which is also observed in a subset of various LSDs when presenting with parkinsonism. Mutations in LRRK2 are associated with either the presence or absence of LB pathology, possibly modulated by additional genetic variations in LSD genes. Yellow, intracellular α-synuclein oligomers.

Current criteria for the pathological diagnosis of sporadic PD include neuronal loss in the SNpc accompanied by LB pathology. In support of this association, there are familial PD cases where LB pathology is consistently present, including α-synuclein-related, or glucocerebrosidase (GBA)-related parkinsonism. Pathogenic missense mutations in α-synuclein, as well as triplication of the α-synuclein locus, cause autosomal-dominant PD with nearly complete penetrance, whilst duplication of the α-synuclein locus causes late-onset PD with reduced (30–50%) penetrance and variable clinical presentations, even within families (Book et al., 2018). Thus, α-synuclein dosage seems to be important, but may not be the only contributor to disease manifestation. Neuropathological examinations of clinically affected patients due to either α-synuclein mutations or multiplications reveal prominent cell loss in the SNpc, associated with severe LB pathology in all cases (Schneider and Alcalay, 2017; Figure 2). Where analyzed, around 50% of cases also display tau pathology, which is similar to the percentage of tau pathology found in sporadic PD cases (Schneider and Alcalay, 2017; Zhang et al., 2018).

Homozygous mutations in the GBA gene, which encodes glucocerebrosidase (GCase), a lysosomal enzyme involved in the metabolism of glycosphingolipids, cause Gaucher disease, the most common autosomal-recessive lysosomal storage disease (LSD). Heterozygous GBA mutations are the strongest genetic risk factor for sporadic PD, and can also increase risk for other α-synucleinopathies. Reduced GBA activity *per se* is not a necessary cause of PD, since most patients heterozygous or homozygous for GBA mutations never develop the disease (Do et al., 2019). However, LB pathology is prominently observed in virtually all GBA-heterozygous mutation carriers manifesting PD (Figure 2), and where analyzed, is accompanied by tau pathology (Schneider and Alcalay, 2017). In addition to heterozygous GBA carriers, the risk of developing PD is also increased in various other LSDs due to mutations in different enzymes which metabolize lipids within lysosomes (Robak et al., 2017). Importantly, in the brains of those patients who develop parkinsonism, the consequent accumulation of several distinct lipids is associated with α-synuclein pathology (Figure 2), as seen in Niemann-Pick disease type C1, Fabry’s disease, Krabbe’s disease, or Sandhoff disease, as well as in several rodent models carrying mutations in such LSD genes (Saito et al., 2004; Suzuki et al., 2007; Shachar et al., 2011; Nelson et al., 2014; Smith et al., 2014).

However, and perhaps surprisingly, not all PD-related gene mutations present with α-synuclein pathology. An increasing amount of evidence from LRRK2 patients indicates that PD diagnosis can be associated with a lack of LB-containing neuropathology (Schneider and Alcalay, 2017). Whilst all LRRK2-PD patients display neuronal loss in the SNpc, around 50% of patients do not show any apparent LB pathology, with variable tau pathology in most cases (Poulopoulos et al., 2012; Kalia et al., 2015; Schneider and Alcalay, 2017; Agin-Liebes et al., 2019; Henderson et al., 2019; Kalia, 2019). The absence of LB pathology is observed in G2019S LRRK2-PD carriers, as well as in carriers of the highly penetrant LRRK2 mutations such as R1441C/G, Y1699C, or I2020T (Schneider and Alcalay, 2017). Consistent with these neuropathological findings, only around 40% of G2019S LRRK2-PD patients display increased α-synuclein aggregates in the cerebrospinal fluid (CSF), in comparison with around 90% of sporadic cases (Garrido et al., 2019). Thus, LRRK2-related PD can be grouped into two classes, one with and one without LB pathology (Figure 2), both accompanied by DA cell loss in the SNpc.

The underlying reason for this remains unknown, but may relate to the presence of additional cellular alteration(s) present in the LB-positive LRRK2-PD patients. Genome-wide association studies (GWAS) have established that LSD gene variants increase risk of developing PD (Foo et al., 2013; Gan-Or et al., 2013; Klemann et al., 2017; Robak et al., 2017; Billingsley et al., 2018). Strikingly, a significant percentage of sporadic PD cases carried one or multiple putative pathogenic variants in distinct LSD genes (Robak et al., 2017). Thus, the LRRK2-PD patients who do display LB pathology may carry additional pathogenic LSD gene variants, with the concomitant lipid accumulation causing α-synuclein aggregation (Figure 2). In support of this possibility, a recent study employing a small set of G2019S LRRK2 patient-derived fibroblasts and iPSC-derived DA neurons revealed a decrease in GCase activity as compared to healthy control cells (Ysselstein et al., 2019). Such reduced GCase activity may play a role as disease modifier, and it will be important to assess GCase activity from a larger set of LRRK2 PD patient-derived cells, and determine whether the decrease in GCase activity correlates with a detectable increase in the lipids which serve as substrates for the enzyme, which may then be able to trigger α-synuclein accumulation.

Glucocerebrosidase is a lysosomal enzyme involved in the metabolism of glycosphingolipids. Loss of GCase enzyme function causes cellular accumulation of glycosphingolipids such as glucosylsphingosine and glucosylceramide, as well as related metabolites and cholesterol (Walkley and Vanier, 2009; Xu et al., 2010). A secondary accumulation of such glycosphingolipids is also observed in several other LSDs (Xu et al., 2010), highlighting the possibility that increased glycosphingolipid levels may trigger α-synuclein oligomerization in the brains of those LSD patients. Bis(monoacylglycero)phosphate (BMP) is a negatively charged glycerophospholipid specifically found in endosomal and lysosomal membranes and thought to play a role in glycosphingolipid degradation and cholesterol transport (Akgoc et al., 2015). Secondary accumulation of BMP has also been reported in several LSDs including Gaucher disease, mucopolysaccharidosis, Niemann-Pick disease, Fabry disease, and gangliosidoses (Vanier, 1983; Käkelä et al., 2003; Hobert and Dawson, 2007; Hein et al., 2013; Akgoc et al., 2015). Interestingly, recent studies have reported an increase in BMP levels in the urine from LRRK2 mutation carriers as compared to non-carriers, and slightly higher BMP levels in those LRRK2 carriers with PD as compared to those without disease (Alcalay et al., 2020). In the future, it will be important to determine whether increased BMP levels correlate with an increase in α-synuclein aggregates in the CSF of those LRRK2 mutation carriers (Garrido et al., 2019). In either case, these data are consistent with the notion of altered lipid levels also in LRRK2-PD patients.

In sum, and at least with respect to LRRK2-related PD, current findings indicate that LBs are neither necessary nor sufficient for cell loss and the clinical expression of parkinsonism. Similarly, tau pathology *per se* may not be the major driving force at least in some cases, as LRRK2-related cell death can occur in the absence of LBs and the presence of a very low burden of tau pathology in the midbrain (Agin-Liebes et al., 2019; Henderson et al., 2019). Finally, the present data indicate that the formation of LBs occurs in a manner independent of pathogenic LRRK2 kinase activity, but possibly mediated by altered lipid metabolism.

## Alpha-Synuclein and PD

Alpha-synuclein is a small, natively unfolded protein which plays an important role in regulating synaptic vesicle release (Murphy et al., 2000; Burré et al., 2010). It is prone to aggregation, giving rise to oligomeric, or protofibrillar structures. Elevated levels of α-synuclein oligomers are observed in the CSF of sporadic PD patients as compared to control subjects (Tokuda et al., 2010; Park et al., 2011), supporting their role in PD pathogenesis. Alpha-synuclein oligomers comprise a very heterogeneous population, and not all of them are toxic to cells. However, for reasons that have not been fully elucidated, certain oligomeric species can be highly toxic, and can disrupt presynaptic transmission, the functioning of several organelles as well as proper protein degradation pathways (Ingelsson, 2016; Bridi and Hirth, 2018). The oligomerization of α-synuclein precedes its aggregation into mature LBs, which as such may play a cytoprotective role.

Alpha-synuclein can act as a prion protein, spreading throughout the nervous system. Indeed, different studies have found LBs in fetal grafted neurons in PD patients (Kordower et al., 2008; Li et al., 2008). Alpha-synuclein oligomers can be released as free-floating proteins or via exosomes. They can then infect adjacent, healthy neurons, where they can act as a seed to induce intracellular α-synuclein accumulation, thereby further contributing to disease progression (Danzer et al., 2009, 2012). In addition, innate-immune astrocytes and microglia can take up α-synuclein oligomers, which may cause multiple proinflammatory changes which are neurotoxic as well. For example, microglial uptake of α-synuclein has been shown to induce the activation of NADPH oxidase, leading to elevated ROS production and contributing to tissue damage over time (Zhang W. et al., 2005).

According to the Braak hypothesis, PD progresses across synaptically connected neuroanatomical locations in the brain, with the appearance of LBs in distinct areas reflecting the distinct clinical manifestations (Braak et al., 2003). However, only around 50% of PD patients have patterns of LB pathology consistent with the Braak staging system (Halliday et al., 2012; Surmeier et al., 2017), and some sporadic (Berg et al., 2014) as well as familial cases (see above) have no discernible LBs at all. It remains possible that these patients are negative for LBs, but display the presence of toxic, oligomeric α-synuclein species. To address this possibility, postmortem material from LRRK2-PD patients negative for LB could be analyzed for the presence of oligomeric forms of α-synuclein, for example employing a proximity ligation assay to detect such α-synuclein species (Roberts et al., 2015). Alternatively, α-synuclein aggregation may not mediate cell death, at least in LB-negative PD patients. Rather than via a propagated pathogen, cell death may be triggered by cell-autonomous or regionally autonomous mechanisms unrelated to protein aggregation (Surmeier et al., 2017).

## Mechanisms Underlying α-Synuclein Oligomerization: a Hypothesis

Whilst point mutations in α-synuclein have been shown to promote increased formation of oligomers *in vitro* (Conway et al., 2000), the molecular mechanism(s) responsible for the formation of wildtype α-synuclein oligomers are less clear. The enhanced oxidative stress inherent to DA neurons in the SNpc may increase its propensity to oligomerize (Sherer et al., 2003), and various post-translational modifications such as nitration or phosphorylation have been reported to enhance oligomerization (Giasson et al., 2000; Anderson et al., 2006; Liu et al., 2011; Follmer et al., 2015).

In addition, impaired degradation may lead to the formation of oligomeric α-synuclein species. The ubiquitin-proteasome system and the autophagy-lysosomal pathway are the two major systems mediating protein degradation in eukaryotic cells. Whilst the proteasome, a multi-subunit protease complex which selectively degrades ubiquitin-tagged proteins, may contribute to the degradation of a proportion of α-synuclein species, most studies indicate that the major route for the degradation is via the autophagy-lysosomal pathway (Stefanis et al., 2019). Different types of autophagy have been described, including chaperone-mediated autophagy (CMA), macroautophagy and microautophagy, and all have been implicated in the degradation of distinct α-synuclein species. CMA is the process by which substrate proteins are selectively targeted to lysosomes and translocated into their lumen through a coordinated action of chaperones and LAMP2A, which translocates substrates directly into the lysosomal lumen (Cuervo, 2010; Cuervo and Wong, 2013). Endogenous wildtype α-synuclein has been shown to be efficiently degraded by CMA (Cuervo et al., 2004), and inhibition of this pathway leads to the formation of α-synuclein oligomers, perhaps secondary to increasing α-synuclein levels (Vogiatzi et al., 2008). In contrast, pathogenic mutant α-synuclein, as well as dopamine-modified, or oligomerized α-synuclein species are poor CMA substrates (Cuervo et al., 2004; Martinez-Vicente et al., 2008). Whilst a role for macroautophagy in the clearance of α-synuclein oligomers is supported by various studies (Webb et al., 2003; Spencer et al., 2009; Yu et al., 2009), others have questioned its relevance. For example, distinct compounds which inhibit lysosomal degradation were found to increase α-synuclein oligomers, but inhibition of macroautophagy was without effect (Lee et al., 2004). Similarly, genetic inhibition of macroautophagy in a rodent model did not lead to detectable α-synuclein aggregates (Ahmed et al., 2012). Thus, it is tempting to speculate that in addition to macroautophagy, at least a portion of α-synuclein oligomers may be degraded via microautophagy.

In mammalian cells, microautophagy occurs in late endosomes/multivesicular bodies (LE/MVB) rather than in lysosomes, and has been termed endosomal microautophagy (eMI; Sahu et al., 2011). This process allows for bulk degradation of small protein oligomers, lipid droplets, mitochondria, or even portions of the nucleus (Roberts et al., 2003; Lemasters, 2014; Seo et al., 2017; Caballero et al., 2018). Cargo is bound to the LE membrane, and trapped into intraluminal vesicles dependent on components of the ESCRT complex, a protein complex required for MVB formation, but independent of LAMP2A, the CMA receptor (Sahu et al., 2011; Mukherjee et al., 2016; Tekirdag and Cuervo, 2018). Some substrates can be directly degraded in LE, whilst others are degraded in the lysosome upon LE-lysosomal fusion (Sahu et al., 2011). In the future, it will be important to obtain direct experimental evidence for the possible relationship between eMI and PD. However, a role for eMI in α-synuclein oligomer degradation is consistent with its localization to the membrane of intraluminal vesicles of LE/MVBs in axons and cell bodies as determined by correlative light and electron microscopy in neurons overexpressing the tagged protein (Boassa et al., 2013). Importantly, eMI is regulated by direct alterations in the lipid composition of the LE membrane, specifically by lipid raft-like regions rich in sphingolipids and cholesterol (Tsuji et al., 2017). Thus, alterations in the lipid composition of LE membranes mediated by GBA and several other LSDs may cause deficits in eMI-mediated degradation of oligomeric α-synuclein species.

Finally, the lipid composition of the membrane may directly contribute to α-synuclein oligomerization independently of effects on protein degradation. Alpha-synuclein monomers are known to bind to synthetic lipid membranes which causes them to adopt an α-helical conformation prone to forming different types of oligomers (Segrest et al., 1992; Davidson et al., 1998; Leng et al., 2001). The membrane lipid composition seems to play an important role, with α-synuclein showing preference for binding to lipid rafts (Leng et al., 2001; Cole et al., 2002; Fortin et al., 2004), and recent studies show that the sphingolipid glycosylceramide can trigger α-synuclein oligomerization (Zunke et al., 2018).

**FIGURE 3:**
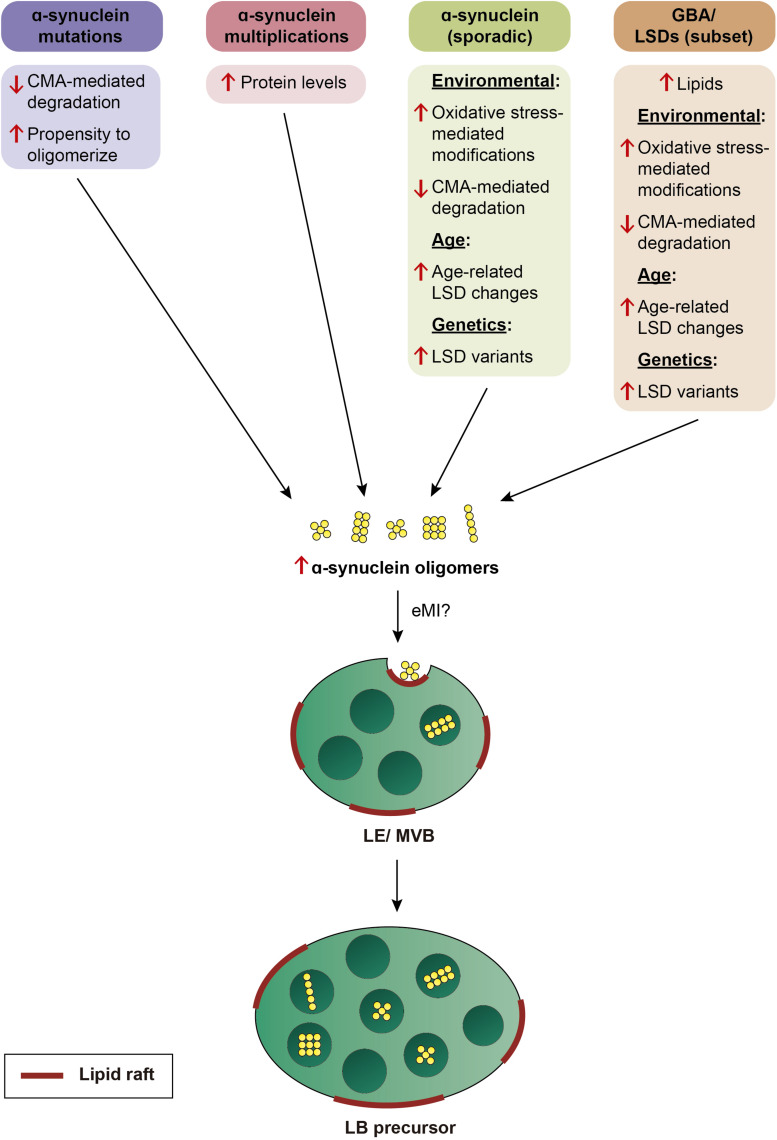
Model by which various genetic alterations cause α-synuclein oligomerization and eventual LB pathology. Mutations in α-synuclein cause oligomerization due to an enhanced propensity of the mutant protein to oligomerize, and/or due to its impaired turnover by CMA. Multiplication of the α-synuclein locus causes oligomerization over time due to an increase in protein levels. In sporadic PD, α-synuclein oligomerization is triggered either by environmental factors, resulting in oxidative stress-mediated posttranslational modifications which interfere with its turnover by CMA, or by age-related or genetic alterations in LSD enzyme activity which result in changes in the membrane lipid composition. In a subset of LSDs including mutations in GBA, oligomerization of α-synuclein is triggered by altered lipid composition in conjunction with additional environmental, age-dependent, or genetic alterations. In all cases, α-synuclein oligomers may be inefficiently cleared by eMI, causing a toxic buildup of cytosolic oligomers over time. The eMI-mediated internalization of α-synuclein oligomers at the LE/MVB may cause the formation of LB precursors over time.

In sum, we propose that α-synuclein aggregates may be a common neuropathological readout, but due to distinct underlying mechanisms (Figure 3). In familial PD cases due to α-synuclein mutations, it may be due to an enhanced propensity of the mutant protein to oligomerize, and/or impaired CMA-mediated turnover. In PD cases due to α-synuclein multiplications, oligomerization may occur as a result of increased protein levels. In PD cases due to mutations in GBA, increased spingolipids may directly trigger α-synuclein binding and oligomerisation, and/or indirectly cause an accumulation of oligomers due to impaired eMI mediated by alterations in the raft-like properties of the LE/MVB. Lipid perturbations may be the main driver also in sporadic PD cases, and due to additional genetic contributions (see above), and/or manifesting in an age-dependent manner, since decreased GBA activity and increased glycosphingolipid levels have been described in the brain with increasing age (Rocha et al., 2015; Hallett et al., 2018). In all cases, the limited capacity of eMI to sequester increasingly abundant oligomers may cause their toxic cellular buildup over time, with oligomer-laden LE/MVB reflecting LB precursors. A LE/MVB-related mechanism for the biogenesis of LBs is consistent with recent ultrastructural studies revealing that LBs from sporadic PD patients are surrounded by a membrane, and comprised of intraluminal α-synuclein along with abundant intraluminal vesicular structures, membrane fragments and dysmorphic organelles (Shahmoradian et al., 2019), a topology very reminiscent of a LE/MVB with its surrounding membrane and intraluminal vesicles.

## Mechanisms Underlying LRRK2-Related PD

Independent studies in different model systems using different and complementary experimental approaches indicate that LRRK2 impairs macroautophagy (Manzoni, 2017; Cogo et al., 2020). Pathogenic LRRK2 overexpression results in an accumulation of autophagosomes in different cell types, including SH-SY5Y and HEK293 cells (Plowey et al., 2008; Gómez-Suaga et al., 2012). Furthermore, data from patient-derived fibroblasts indicate that mutant LRRK2 causes an increase in autophagosome numbers, with an impaired response to starvation-induced autophagy (Bravo-San Pedro et al., 2013; Manzoni et al., 2013). Similarly, iPSC-derived DA neurons from G2019S LRRK2-PD patients display an increase in autophagosome numbers due to a decrease in autophagosome clearance (Sánchez-Danés et al., 2012). This is consistent with our study reporting that the increase in autophagosome numbers is paralleled by a decrease in the number of acidic lysosomes, which is expected to cause lysosomal degradative deficits (Gómez-Suaga et al., 2012). Similarly, pathogenic LRRK2 has been consistently reported to cause impaired mitophagy in various cell types, likely due to limited lysosomal clearance of mitochondria (Cherra et al., 2013; Hsieh et al., 2016; Verma et al., 2017; Bonello et al., 2019; Korecka et al., 2019; Walter et al., 2019). Altogether, these data indicate that mutant LRRK2 causes a deficit in lysosomal functioning, thereby impairing the autophagic clearance of protein aggregates and dysfunctional organelles.

Indeed, multiple independent studies indicate that disease-associated LRRK2 variants alter lysosomal biology, even though not due to alterations in protein expression levels as assessed from G2019S knockin tissue, in contrast to tissue obtained from LRRK2-deficient mice (Pellegrini et al., 2018). Different lysosomal readouts have been analyzed, including altered lysosome localization, number, size or luminal pH, with only few studies directly assessing the potential effects of those alterations on lysosomal protein degradation capacity. Studies in a Drosophila model overexpressing G2019S LRRK2 indicate that pathogenic LRRK2 promotes the perinuclear clustering of lysosomes in a manner dependent on Rab7a and microtubules (Dodson et al., 2012). A decrease in lysosome number accompanied by an increase in lysosome size, similar to the phenotype of enhanced perinuclear lysosomal clustering observed in Drosophila, has also been reported in cultured astrocytes derived from transgenic mice overexpressing G2019S LRRK2 (Henry et al., 2015). Importantly, these morphological alterations were shown to be accompanied by impaired lysosomal degradation (Henry et al., 2015). Lysosomal structural alterations, namely swollen lysosomes, have also been described at the electron microscopy level in cultured neurons overexpressing G2019S LRRK2 (MacLeod et al., 2006). In cellular systems expressing endogenous amounts of pathogenic LRRK2, lysosomal phenotypes have been reported as well. For example, increased lysosome size and perinuclear clustering is observed in fibroblasts from G2019S LRRK2 PD patients as compared to healthy controls (Hockey et al., 2015). Interestingly, both readouts are reversed upon inhibiting the activity of TPC2, an endolysosomal ion channel implicated in calcium signaling from acidic organelles (Hockey et al., 2015), consistent with our findings that pathogenic LRRK2-mediated autophagic deficits are reverted upon TPC2 inhibition (Gómez-Suaga et al., 2012). Similarly, a recent study reported increased lysosomal pH, decreased lysosomal protein degradation capacity as well as altered lysosomal calcium dynamics in cortical neurons from BAC transgenic rats expressing R1441C LRRK2 (Wallings et al., 2019). Modest but significant lysosomal alterations have also been reported in cultured cortical neurons from G2019S knockin mice, with an increase in lysosome number and a decrease in lysosomal acidity, accompanied by increased insoluble α-synuclein and enhanced α-synuclein secretion (Schapansky et al., 2018). Thus, a picture is emerging whereby pathogenic LRRK2 causes lysosomal structural alterations which are paralleled by impaired lysosomal protein degradation capacity, increased intralysosomal pH and altered lysosomal calcium signaling, all of which may contribute to PD pathogenesis.

## The LRRK2-RAB Axis Underlying Endolysosomal Deficits

**FIGURE 4:**
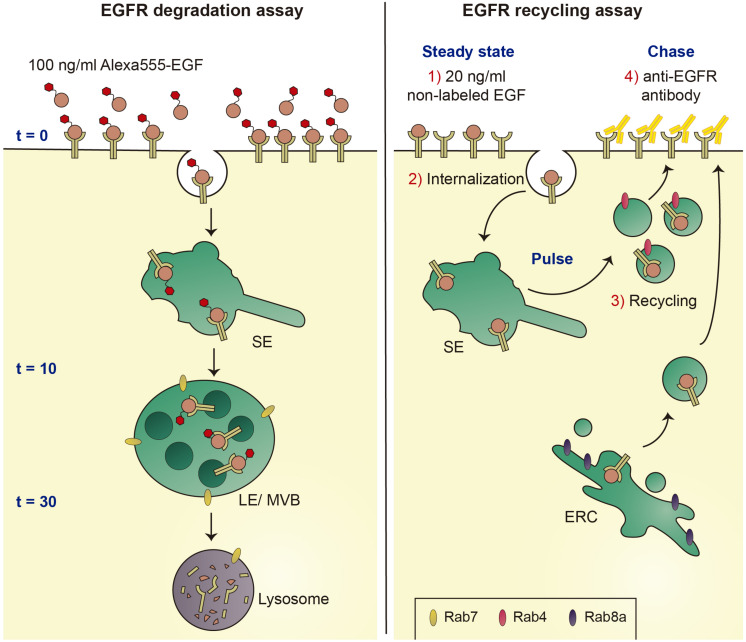
Schematics of EGFR degradation and recycling assays. Left: Under conditions of high ligand concentrations, fluorescent EGF is internalized by endocytosis and sorted to lysosomes for degradation. EGFR surface availability can be assessed upon binding of Alexa555-EGF to cells at 4°C (*t* = 0 min), and endocytic trafficking and degradation can be followed by quantification of endocytosed fluorescent EGF over time (*t* = 10 min and *t* = 30 min). Right: under conditions of low ligand concentrations, the EGFR is endocytosed and recycled to the cell surface using either a fast recycling pathway dependent on Rab4, or a slow recycling pathway via the ERC and dependent on Rab8a. Non-fluorescent EGF is bound to cells at 4°C, cells are shifted to 37°C to allow for EGFR internalization (pulse), followed by a chase for various time points to assess recycling rates back to the cell surface (chase). Reappearance of the EGFR at the cell surface is detected using an antibody against the extracellular domain of the EGFR (yellow) in the absence of cell permeabilization. SE, sorting endosome; LE/MVB, late endosome/multivesicular body; and ERC, early recycling compartment.

To dissect the mechanism(s) underlying the endolysosomal alterations caused by pathogenic LRRK2, we employed the well-established epidermal growth factor receptor (EGFR) trafficking and degradation assays. Under high concentrations of ligand, the receptor undergoes a purely degradative trafficking route, whilst low ligand concentrations favor receptor recycling, allowing us to probe for effects of pathogenic LRRK2 on both trafficking routes (Figure 4). Pathogenic LRRK2 expression was found to cause a deficit in EGFR degradation which was reversed upon addition of distinct LRRK2 kinase inhibitors or when overexpressing active Rab7a (Gómez-Suaga et al., 2014). The deficit in EGFR trafficking and degradation correlated with the appearance of Rab7a-positive tubular structures, which could be observed in cells expressing pathogenic LRRK2 as well as in fibroblasts from PD patients carrying the G2019S LRRK2 mutation as compared to healthy controls. In both cases, the trafficking deficit and formation of Rab7a-positive tubules were associated with a significant decrease in the levels of active Rab7a as measured using biochemical pulldown assays (Gómez-Suaga et al., 2014).

Whilst Rab7a is not required for the trafficking to LE/MVB nor the formation of intraluminal vesicles at the LE/MVB, it is required for transfer of cargo from LE/MVB to the lysosome (Vanlandingham and Ceresa, 2009), and known to regulate endolysosomal positioning, retromer-mediated trafficking, lysosome reformation, and autophagosome maturation (Bucci et al., 2000; Jordens et al., 2001; Jäger et al., 2004; Ceresa and Bahr, 2006; Rojas et al., 2008; Yu et al., 2010). Thus, pathogenic LRRK2 may interfere with these various endolysosomal trafficking steps via decreasing Rab7a activity. Even though ubiquitously expressed, missense mutations in Rab7 are known to cause Charcot-Marie-Tooth type 2B disease, a peripheral neuropathy (Verhoeven et al., 2003; Houlden et al., 2004; Meggouh et al., 2006; Bucci and De Luca, 2012). In addition, in a Drosophila model, loss of Rab7 in neurons causes adult-onset degeneration beginning with a loss of synaptic function (Cherry et al., 2013), warranting further *in vivo* studies to address the role of Rab7 in neurodegeneration and specifically in LRRK2-related PD pathogenesis.

The mechanism by which pathogenic LRRK2 causes a decrease in Rab7a activity remains unknown, and various scenarios are possible. LRRK2 phosphorylates a subset of Rab proteins on a conserved residue within the switch II domain, which is implicated in GDP/GTP exchange as well as in interactions with various regulatory and effector proteins (Steger et al., 2016, 2017). A phosphomimetic Rab8a mutant displays impaired binding to GDP dissociation inhibitor 1/2 (GDI1/2), which is essential to target and extract the Rab protein from the membrane, and also to Rabin8, the guanine nucleotide exchange factor (GEF) required to activate the protein (Steger et al., 2016; Madero-Pérez et al., 2018). Whilst such binding differences are less pronounced with endogenously phosphorylated Rab8a (Steger et al., 2017), these biochemical studies nevertheless are consistent with the idea that LRRK2-mediated phosphorylation of Rab8a causes its inactivation (Steger et al., 2016). By analogy, LRRK2-mediated phosphorylation of Rab7a may contribute to its inactivation, even though current evidence suggests that Rab7a is not a LRRK2 kinase substrate either *in vitro* or *in vivo* (Steger et al., 2016, 2017; Rivero-Ríos et al., 2019), in contrast to its reported phosphorylation by the related kinase LRRK1 (Hanafusa et al., 2019). Alternatively, LRRK2 may regulate Rab7a protein levels, even though the absence of detectable differences in protein levels from G2019S LRRK2 PD patients as compared to healthy controls makes this scenario unlikely (Gómez-Suaga et al., 2014). A third scenario includes LRRK2-mediated alterations in the functioning of at least some of the Rab proteins which are LRRK2 kinase substrates, thereby causing downstream deficits in Rab7a activity.

**FIGURE 5:**
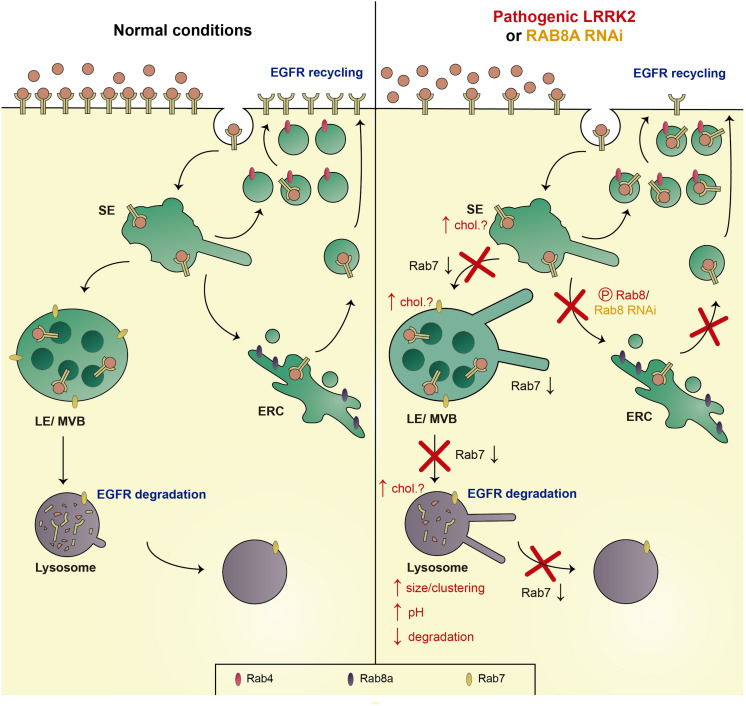
Schematics of trafficking deficits mediated by pathogenic LRRK2 or knockdown of Rab8a. Left: Under normal conditions, the EGFR is either endocytosed and sorted to LE/MVB and the lysosome for degradation (EGFR degradation), or recycled back to the membrane via either a fast (Rab4-dependent) or a slow recycling pathway (Rab8a-dependent; EGFR recycling). Right: Pathogenic LRRK2 (via phosphorylation of Rab8a), or knockdown of Rab8a causes a decrease in active Rab7a, causing a deficit in trafficking from the SE to the LE/MVB, from the LE/MVB to the lysosome, as well as a deficit in lysosome reformation, associated with an increase in the size/clustering of lysosomes, an increase in lysosomal pH, and a decrease in lysosomal degradation. In addition, the LRRK2-mediated phosphorylation of Rab8a, or knockdown of Rab8a, causes a deficit in trafficking to/from the ERC, with a resultant accumulation of the EGFR in a Rab4-positive recycling compartment. Speculative alterations in cholesterol levels across the endocytic pathway may further contribute to the pathogenic LRRK2-mediated deficits (chol?). SE, sorting endosome; LE/MVB, late endosome/multivesicular body; and ERC, early recycling compartment.

The most prominent LRRK2 kinase substrates include Rab8a and Rab10 (Steger et al., 2016, 2017). Rab8a is localized to the Golgi and a tubular early recycling compartment, and is known to regulate post-Golgi exocytic membrane trafficking, and endocytic recycling steps (Hattula et al., 2006; Peränen, 2011; Vaibhava et al., 2012; Kim et al., 2017). Since Rab8a has also been described to modulate endolysosomal trafficking events (Braun et al., 2015), we probed for a possible link between alterations in Rab8a and the endolysosomal degradative trafficking steps of the EGFR which are impaired by G2019S LRRK2 (Rivero-Ríos et al., 2019). Expression of active Rab8a, or upregulation of the Rab11-Rabin8 cascade known to activate Rab8a, rescued the G2019S LRRK2-mediated trafficking deficits. Conversely, knockdown of Rab8a impaired endolysosomal trafficking and degradation of the EGFR, which could be rescued upon expression of active Rab7a. In sum, either expression of G2019S LRRK2 or knockdown of Rab8a cause a decrease in the activity of endogenous Rab7a, accompanied by identical endolysosomal trafficking deficits (Figure 5; Gómez-Suaga et al., 2014; Rivero-Ríos et al., 2019).

In the future, it will be important to determine the link between other kinase substrates such as Rab10 and the LRRK2-mediated endolysosomal trafficking deficits. In addition, further studies are warranted to determine how downregulation of Rab8a, either by RNAi, or by LRRK2-mediated phosphorylation, causes a decrease in Rab7a activity. Crosstalk between these two Rab proteins may be mediated by a competition for shared GEFs, even though the currently identified GEFs for Rab8a and Rab7a are distinct (Burton et al., 1994; Hattula et al., 2002; Nordmann et al., 2010; Yoshimura et al., 2010; Yasuda et al., 2016; Devergne et al., 2017). Alternatively, the two Rab proteins may compete for GTPase-activating proteins (GAPs), which display a certain degree of promiscuity and little reported correlation between their Rab binding and Rab-GAP activities (Itoh et al., 2006; Fukuda, 2011). TBC1D15 has been described as a GAP for Rab7a (Zhang X. M. et al., 2005; Peralta et al., 2010). TBC1D15 expression causes the same endolysosomal trafficking deficits as pathogenic LRRK2, which are rescued by GTP-locked Rab7a expression and correlate with a decrease in endogenous Rab7a activity (Gómez-Suaga et al., 2014). Interestingly, TBC1D15 has been described to preferentially interact with phospho-deficient as compared to phospho-mimetic Rab8a (Steger et al., 2016). Thus, the LRRK2-mediated phosphorylation of Rab8a may cause a decreased interaction with TBC1D15, allowing it to act on Rab7a, similar to what has been recently described for the retromer-mediated regulation of Rab7a activity via TBC1D5 (Jimenez-Orgaz et al., 2018).

Another attractive hypothesis relates to a possible regulation of Rab7a activity by distinct lipids. Interestingly, several studies indicate that Rab7a activity is regulated by cholesterol (Choudhury et al., 2002, 2004; Lebrand et al., 2002; Pagano, 2003). In many LSDs, the endolysosomal accumulation of glycolipids is associated with the secondary accumulation of cholesterol (Pagano, 2003), and overexpression of Rab7a has been reported to correct such accumulation (Choudhury et al., 2002). In addition, increased endolysosomal cholesterol impairs the GDI-mediated extraction of Rab7a from the membrane, with possible consequences for the balance between active and inactive Rab7a (Lebrand et al., 2002). Conversely, a role for Rab8a in cholesterol trafficking has been described as well. The endolysosomal cholesterol and sphingolipid deposition in LSD fibroblasts from Niemann-Pick type C disease is rescued by overexpression of Rab8a, whilst depletion of Rab8a from wildtype fibroblasts causes an accumulation of endolysosomal cholesterol (Linder et al., 2007). Finally, expression of pathogenic G2019S LRRK2 or loss of Rab8a causes an accumulation of the EGFR in a Rab4-positive endocytic compartment (Rivero-Ríos et al., 2019), and a cholesterol-mediated impairment of the Rab4-dependent recycling pathway has been previously described (Choudhury et al., 2004). Thus, further studies are warranted to probe for a link between LRRK2, Rab8a and altered intracellular cholesterol levels, which may underlie the various reported trafficking deficits along the endosomal/endolysosomal system via direct inactivation of Rab7a (Figure 5).

Irrespective of the precise mechanism(s) underlying Rab7a inactivation mediated by pathogenic LRRK2, the consequences may involve a loss of lysosomal identity and function, further exacerbated in the context of lysosomal stress. Our EGFR trafficking studies were performed in the presence of serum starvation which favors high flux through the endocytic system, which is accompanied by the stimulation of lysosome reformation. This process is regulated by Rab7a, and involves the generation of LAMP1-positive tubules which pinch off from the lysosomal compartment (Yu et al., 2010). Impaired lysosome reformation, as evidenced by excess lysosomal tubulation, will lead to an eventual loss of lysosome identity and function, since the tubule-derived vesicles initially contain lysosomal membrane components but are devoid of acidic pH, or intraluminal proteases (Yu et al., 2010). Interestingly, upon induction of lysosomal tubule formation, these structures were reported not to be preserved upon fixation, with staining for lysosomal membrane markers instead revealing clustered lysosomes, measured as an increase in size and concomitant decrease in numbers as compared to control conditions (Sridhar et al., 2013), identical to the structural lysosomal changes observed upon pathogenic LRRK2 expression. Thus, all reported alterations in lysosome morphology and function are consistent with a possible pathogenic LRRK2-mediated impairment of lysosome reformation in a manner mediated by Rab7a.

Additional roles for LRRK2 under conditions of lysosomal stress triggered by distinct lysosomotropic agents have been described. Whilst LRRK2 is largely not localized to lysosomes under normal conditions (Gomez et al., 2019), these agents are able to potently trigger its lysosomal recruitment. For example, prolonged treatment of cells with chloroquine, which becomes protonated and trapped in acidic compartments including LE/MVB and lysosomes, causes endolysosomal enlargement, translocation of endogenous wildtype LRRK2, accumulation of Rab8a and Rab10, and the promotion of lysosome secretion (Eguchi et al., 2018). Whilst these data suggest that wildtype LRRK2 plays a role in lysosomal homeostasis, the effect of pathogenic LRRK2 variants on this readout remains to be determined. On the other hand, recent studies using LLOME, a membranolytic polymer causing lysosomal damage, was described to cause the endolysosomal recruitment of wildtype LRRK2, accumulation and phosphorylation of Rab10 and Rab35, and subsequent recruitment of JIP4 (Bonet-Ponce et al., 2020). Interestingly, this was associated with the formation of JIP4-positive, LAMP1-negative tubules, and was more pronounced when expressing G2019S LRRK2 as compared to wildtype LRRK2 (Bonet-Ponce et al., 2020). Thus, and under conditions of lysosomal stress, the pathogenic LRRK2 kinase-mediated processes may further compromise lysosomal identity and function, even though the pathophysiologically relevant lysosomal stressor(s) remain to be determined.

## Concluding Remarks

**FIGURE 6:**
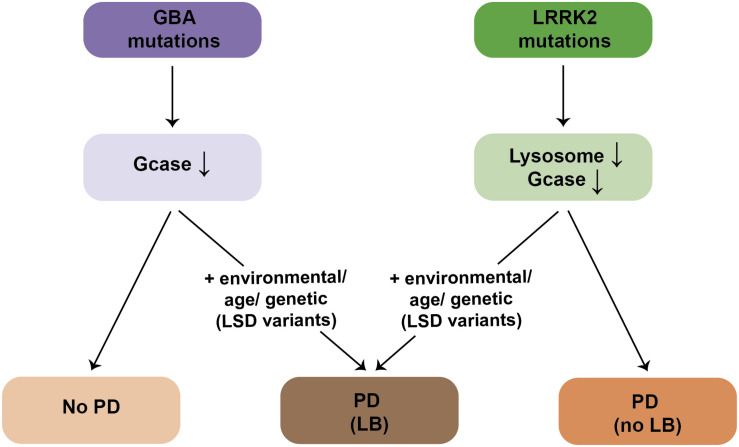
Schematics for LB formation in patients with mutations in GBA or LRRK2. The majority of GBA mutation carriers display a reduction in GCase enzyme activity in the absence of PD. PD manifestation in GBA mutation carriers requires additional environmental, age-related or genetic (possibly other LSD gene variants) triggers, which is accompanied by α-synuclein aggregation/LB formation. Pathogenic LRRK2 mutation carriers display deficits in lysosomal functioning due to deficits in proper endolysosomal trafficking, which is also associated with a decrease in GCase enzyme activity. Deficits in lysosomal functioning cause PD in the absence of LB formation. The presence of LBs in LRRK2-PD patients requires additional environmental, age-related or genetic (possibly other LSD gene variants) triggers.

An increasing amount of evidence indicates that lysosomal deficits are central to PD pathogenesis. In the case of PD due to mutations in LRRK2, structural and functional lysosomal alterations may arise due to impaired lysosome reformation in a manner dependent on Rab7a, with lysosomal identity and function further compromised in the context of additional lysosomal stress. Such LRRK2-mediated lysosomal dysfunction may lead to progressive cellular demise and manifestation of PD symptoms in the absence of LB formation (Figure 6). Lysosomal deficits mediated by pathogenic LRRK2 may not be sufficient to cause α-synuclein aggregation, but may manifest only in the context of additional lysosomal stress. Alternatively, and as proposed here, the mechanisms leading to LB formation in a subset of LRRK2-PD patients may be independent of the LRRK2-mediated lysosomal deficits, and involve α-synuclein oligomer formation triggered by altered lipid composition due to the existence of additional genetic LSD variants in those patients, or due to additional environmental and/or age-related changes (Figure 6). Conversely, patients with mutations in GBA display reduced GCase enzyme activity, but the majority never develop PD. Disease manifestation in GBA carriers may also require additional environmental, genetic or age-related triggers, resulting in lipid alterations, and concomitant α-synuclein aggregation (Figure 6).

The pathogenic LRRK2-mediated lysosomal alterations are evidenced by an increase in lysosomal pH, expected to decrease the activity of various lysosomal proteases. Interestingly, LRRK2 kinase inhibitors were recently shown to cause reacidification of lysosomal pH and a partial correction of cathepsin B activity in GBA-mutant astrocytes (Sanyal et al., 2020). In addition, decreased GCase activity has been described in G2010S LRRK2-PD fibroblasts and iPSC-derived neurons, which was corrected upon LRRK2 kinase inhibitor application or Rab10 expression, and was mimicked upon knockdown of Rab10 (Ysselstein et al., 2019). However, LRRK2 kinase inhibitor treatment or Rab10 expression resulted in a similar increase in GCase activity also in healthy control cells and in cells heterozygous for GBA mutations (Ysselstein et al., 2019), suggesting a mechanism independent of mutation status or disease state, perhaps related to altered endolysosomal membrane trafficking events. Indeed, impaired Golgi-lysosomal transport of GBA enzyme causes a decrease in its activity (Rothaug et al., 2014), such that endolysosomal trafficking deficits may explain the reported decrease in GCase activity in pathogenic LRRK2-expressing cells (Ysselstein et al., 2019; Figure 6). Whilst further work is required to dissect the precise crosstalk between LRRK2 and GBA, these data highlight the importance of the LRRK2-Rab axis in regulating lysosomal homeostasis, possibly relevant beyond LRRK2-related PD. In addition, they indicate that studies addressing the role of Rab function in cell culture and rodent models will be of paramount importance toward understanding PD-related cellular mechanisms, and that modulators of Rab activity may have potential as disease-modifying agents.

## Author Contributions

SH conceived and wrote the manuscript. PR-R generated all artwork. PR-R, MR-L, RF, and YN provided intellectual input for the contents and edited the manuscript.

## Conflict of Interest

The authors declare that the research was conducted in the absence of any commercial or financial relationships that could be construed as a potential conflict of interest. The handling Editor declares a past co-authorship with one of the authors SH.
